# Evaluation of takayasu arteritis with delayed contrast-enhanced MR imaging by a free-breathing 3D IR turbo FLASH

**DOI:** 10.1097/MD.0000000000009284

**Published:** 2017-12-22

**Authors:** Min Liu, Weifang Liu, Haoyuan Li, Xiaoming Shu, Xincao Tao, Zhenguo Zhai

**Affiliations:** a Department of Radiology; b Department of Rheumatology; c Department of Pulmonary and Critical Care Medicine, China-Japan Friendship Hospital, Beijing, China.

**Keywords:** delayed contrast-enhanced MR imaging, magnetic resonance angiography, Takayasu arteritis

## Abstract

The primary aim of our case-control study was to observe delayed contrast-enhanced magnetic resonance imaging (DCE-MRI) in patients with Takayasu arteritis (TA) in comparison with magnetic resonance angiography (MRA). Twenty-seven patients including 15 with active TA and 12 with stable TA who underwent both aortic MRA and DCE-MRI were included. A total of 27 sex- and age-matched healthy volunteers were enrolled as the control group. MRA were obtained with T1WI-volume-interpolated breath-hold examination sequence or fast low-angle shot (FLASH) sequence. DCE-MRI was acquired with a free-breathing three-dimensional inversion recovery Turbo fast low-angle shot (3D IR Turbo FLASH). Neither stenosis nor delayed enhancement of arterial wall was shown in the control group. In patients with stable TA, arterial stenosis was observed on MRA. On DCE-MR, delayed enhancement of arterial walls could be observed in the active TA group but not in the stable TA group or the control group. Stenotic arteries on MRA were comparable in the active TA and stable TA (χ^2^ = 2.70, *P* = .259); however, delayed enhancement of arterial walls in the active-TA group were more than those in the stable group (χ^2^ = 27.00, *P* < .001). Our results suggest that DCE-MRI with the free-breathing 3D IR Turbo FLASH sequence could assess TA and delayed enhancement on DCE-MRI is one characteristics of the active TA.

## 1 Introduction

Takayasu arteritis (TA) is an idiopathic chronic inflammatory disease that primarily affects aorta, its major branches, and the pulmonary arteries. It is characterized by granulomatous inflammation of the arterial wall with marked intimal proliferation and fibrosis of the media and adventitia.^[1]^ Early diagnosis and treatment of TA is important in preventing serious complications.

Conventional angiography, the criterion standard method for initial diagnosis, seems to be replaced with the imaging modalities such as computer tomographic angiography (CTA) or magnetic resonance imaging (MRI) in recent years.^[2–4]^ CTA has been an important role in diagnosis and activity determination of TA^[5]^; however, radiation dose need to be considered. Contrast-enhanced 3-dimensional MR angiography (MRA) can noninvasively assess luminal stenosis or dilation without radiation; however, this method is limited to visualize situation of vessels wall. Delayed contrast-enhanced MR Imaging (DCE-MRI) has demonstrated its value for the detection of vessel wall alterations in TA.^[6,7]^ Delay enhancement of arterial wall on DCE-MRI can be demonstrated with a delay after injection of gadolinium contrast material, which depicts progressive accumulation and delayed washout of contrast medium.

Recently, the free-breathing 3-dimensional inversion recovery turbo fast low-angle shot (3D IR Turbo FLASH) sequence^[8,9]^ was developed to evaluate myocardial fibrosis in form of delayed enhancement in patients with cardiomyopathy. Because inflammation and fibrosis^[10]^ were pathological characteristics of arterial wall of TA, we hypothesized that delayed enhancement could be observed in TA by using a free-breathing navigator-gated 3D IR Turbo FLASH sequence. The purpose of this study is to observe DCE-MRI findings of TA with free-breathing 3D IR Turbo FLASH and compare DCE-MRI with MRA in patients with TA.

## 2 Methods

### 2.1 Participants

This is a case-control observational study. The institutional ethics committee of our hospital approved the study protocol and all participants gave the written informed consent for this study. According to modified version of the Ishikawa diagnostic criteria for TA^[11]^ and the National Institute Health criteria,^[12,13]^ we prospectively included 15 patients with the actively TA from January 2016 to May 2017. Meanwhile, we screened 12 patients with the stable TA between January 2014 and October 2015 from medical charts. Twenty-seven sex- and age-matched healthy volunteers without a history of pulmonary, cardiovascular, or systemic diseases were enrolled as the control group. All participants underwent both aortic MRA and DCE-MRI. Demographic data were collected from all patients ‘medical charts.

### 2.2 Magnetic resonance imaging

All patients underwent MRI on 3.0-T scanner (Tim Trio, Erlangen, Siemens Healthcare, Germany) with a body-flex receiver coil. After scout imaging, MRA were obtained by using coronial and transversal T1WI-volume-interpolated breath-hold examination sequence (repetition time [TR]/echo time [TE] = 3.20/1.23 ms, flip angle = 10°, field of vision (FOV) = 400 × 400 mm, voxel size = 1.8 × 1.3 × 2.5 mm, slice thickness = 2 mm, slices per slab = 60, slabs = 1, matrix = 320 × 224, bandwidth = 560 Hz/pixel, generalized autocalibrating partially parallel acquisitions (GRAPPA) = 2) or by using FLASH sequence (TR/TE = 2.66/0.98 ms, flip angle = 30°, FOV = 500 × 500 mm, voxel size = 1.3 × 1.0 × 2.5 mm, slice thickness = 1.2 mm, slices per slab = 88, slabs = 1, matrix = 512 × 256, bandwidth = 650 Hz/pixel, GRAPPA factor = 2) after injection of gadopentetate dimeglumine (Magnevist, Schering, Berlin, Germany) was applied at 0.2 mmol/kg body weight at a rate of 3 mL/s followed by a 20 to 30 mL normal saline flush from a separate syringe at a rate of 2 mL/s using a power injector (Mallinckrodt, St Louis). After 10 to 15 minutes, DCE-MRI was obtained with a free-breathing 3D IR Turbo FLASH sequence. A crossed pair navigator pulse was used for respiratory gating. This protocol was performed in a transversal slab covering ascending aorta, aortic arch and its branches, and thoracic descending aorta. Typical imaging parameters for 3D IR Turbo FLASH sequence were TR/TE = 2.8/1.05 ms, flip angle = 15^o^, TI = 260 ms, bandwidth = 610 Hz/pixel, number of k-space lines per cardiac cycle 35, data window duration = 117 ms, FOV = 340 × 280 mm, matrix = 256 × 256, slices per slab = 70, slabs = 1, voxel size = 1.3 × 1.3 × 3.0 mm, GRAPPA factor = 2. The acceptable window of navigation was 2.5 mm, and respiratory motion adaptation was used.

### 2.3 Magnetic resonance images analysis

Two radiologists who were blinded to the clinical information independently reviewed MRA and DCE-MRI of each subjects, determined stenosis, and the delayed enhancement of arterial wall. Aorta with its main branches including branchiocephalic, subclavian artery, common carotid, ascending aorta, the aortic arch, descending thoracic aorta, abdominal aorta, and pulmonary artery (PA) were assessed. The luminal situation on MRA is classified into normal, stenosis/occlusion, and aneurismal dilatation. DCE-MRI of arterial wall was classified into the positive and negative delayed enhancement.

### 2.4 Statistical Analysis

Quantitative data were expressed as mean ± standard deviation and/or median value. Comparison of qualitative findings was performed by Chi-square test or Fisher exact test. Comparison of quantitative clinical and MRI findings was performed by Mann-Whitney *U* test. Analysis was performed with MedCalc Statistical Software version 14.8.1 (MedCalc Software, Ostend, Belgium). For all tests, a *P* value <.05 was considered statistically significant.

## 3 Results

### 3.1 Study participants

The clinical information of TA patients is shown in Table 1. TA patients included 1 men and 26 women, with median age of 34 years (range 22–43 years). Healthy control group involved 1 man and 26 women with median age of 34 years (range 23–40 years). Sex and age (*U* = 335.00, *P* = .591) between TA and the healthy group are similar. Red blood cell count (RBC), white blood cell count (WBC), hemoglobin, platelet count, erythrocyte sedimentation rate (ESR), C-reactive protein (CRP) and N-terminal pro-brain natriuretic peptide (NT-proBNP) are shown in Table 2. Compared with the control group, WBC (*U* = 240.0, *P* = .031), platelet (*U* = 178.5, *P* = .001), ESR (U = 130.0, *P* < .001), CRP (*U* = 84.0, *P* < .001), and NT-proBNP (*U* = 78.0, *P* < .001) in TA group increased.

**Table 1:**
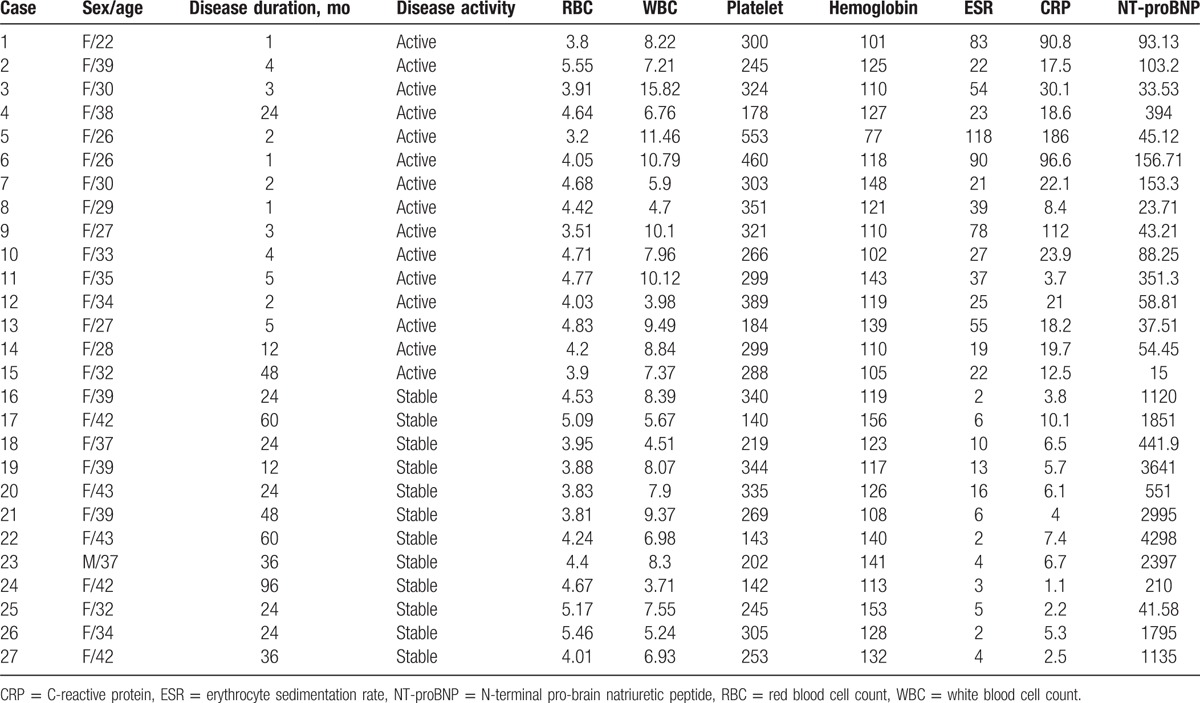
The clinical information of patients with Takayasu arteritis.

**Table 2:**
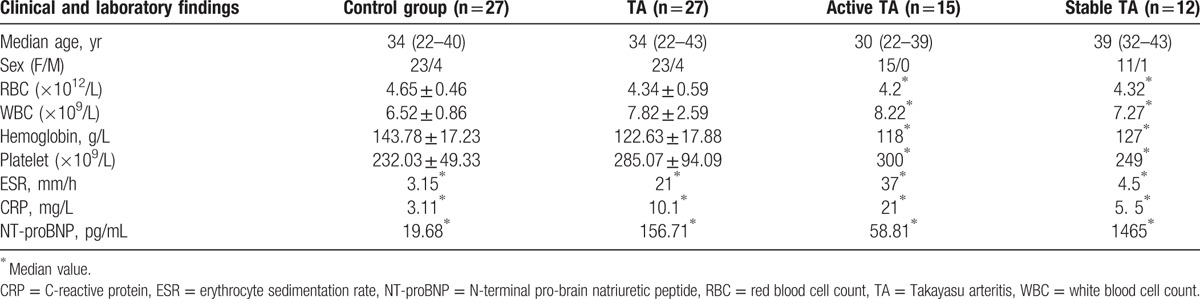
clinical and laboratory findings in the Takayasu arteritis and healthy control.

The active TA group included 15 women with median age of 30 years and the disease duration ranged from 1 to 48 months. The stable TA group involved 1 man and 11 women with median age of 39 years and the disease duration ranged from 12 to 96 months. Table 2 shows age (*U* = 10.5, *P* = .747), RBC (*U* = 84.0, *P* = .770), WBC (*U* = 57.0, *P* = .107), hemoglobin (*U* = 52.5, *P* = .067), and platelet count (*U* = 55.5, *P* = .092) were comparable in the active and stable TA group. ESR (*U* = 31.0, *P* = .003) and CRP (*U* = 14.0, *P* < .001) in the active TA group was significantly increased; however, NT-proBNP significantly increased in the stable group (*U* = 14.5, *P* < .001).

### 3.2 Magnetic resonance angiography and delayed contrast-enhanced magnetic resonance imaging

**Figure 1:**
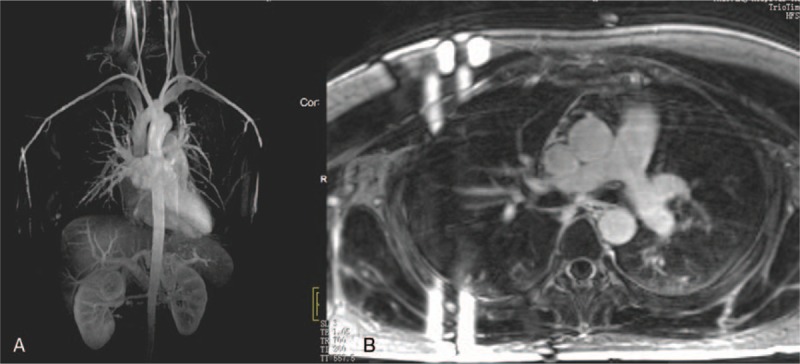
A 22-year-old woman in healthy group. Magnetic resonance angiography (MRA) (A) showed the normal aortic and pulmonary artery. Delayed contrast-enhanced MRI (DCE-MRI) with 3-dimensional inversion recovery Turbo fast low-angle shot (3D IR Turbo FLASH) (B) did not show delay enhancement of arterial wall.

**Figure 2:**
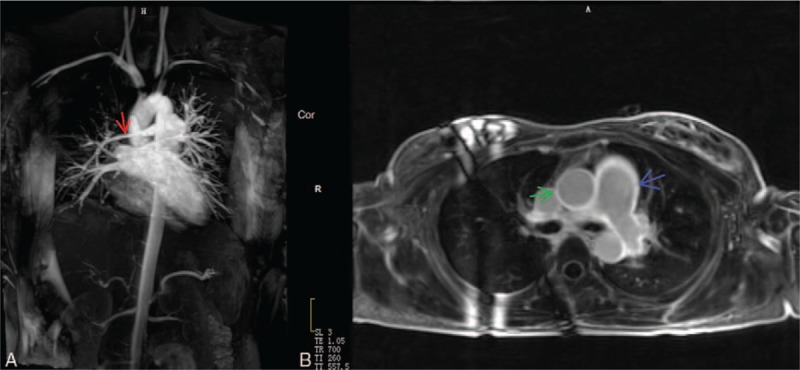
A 22-year-old woman with the active Takayasu arteritis. Magnetic resonance angiography (MRA) (A) showed the stenotic right pulmonary artery (red arrow). Delayed contrast-enhanced magnetic resonance imaging (DCE-MRI) with three-dimensional inversion recovery Turbo fast low-angle shot (3D IR Turbo FLASH) (B) showed delay enhancement in aorta (green arrow) and pulmonary arteries (blue arrow).

**Figure 3:**
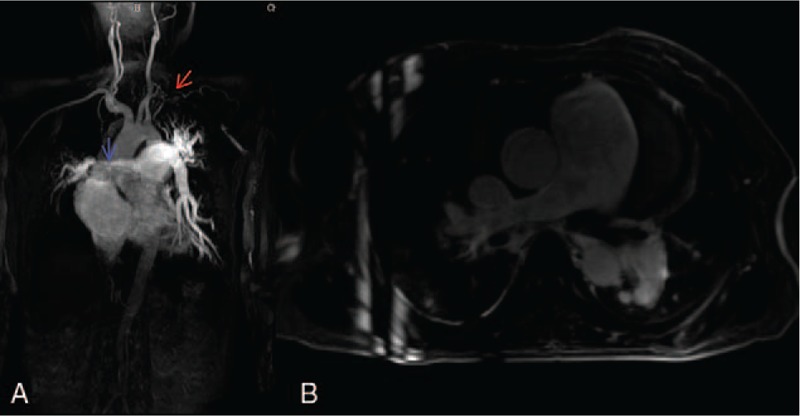
A 32-year-old woman with the stable Takayasu arteritis. Magnetic resonance angiography (MRA) (A) showed the stenotic left subclavian artery (red arrow) and right pulmonary artery (blue arrow). DCE-MRI with three-dimensional inversion recovery Turbo fast low-angle shot (3D IR Turbo FLASH) (B) did not show delay enhancement in the arterial walls.

The stenotic arteries in the TA group are shown in Table 3. In the healthy group, none showed stenotic arteries on MRA (Fig. 1). In active TA group, MRA could show the stenotic arteries (Fig. 2). Five patients showed stenosis in aorta and its branches and 3 cases showed stenosis in PA and its branches, whereas another 7 patients showed stenosis in both aorta and PA and their branches. In the stable TA group, MRA also could demonstrate the stenotic arteries (Fig. 3). Five cases showed stenosis in aorta and its branches, whereas 7 cases showed stenosis in both aorta and PA and their branches. There was no significantly difference of stenotic arteries between the active and stable TA patients (χ^2^ = 2.70, *P* = .259).

**Table 3:**
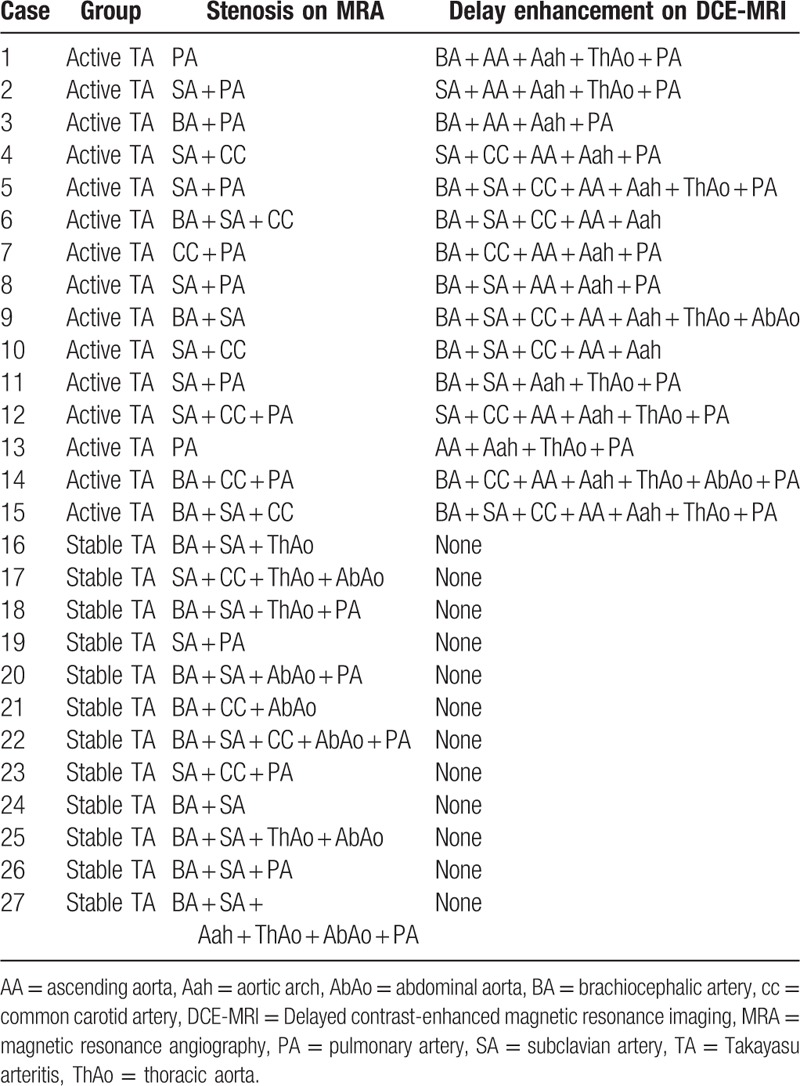
Magnetic resonance angiography and delayed contrast-enhanced magnetic resonance imaging of patients with Takayasu arteritis.

Table 3 shows the delayed enhancement of arterial wall in TA group. In the healthy group, no delayed enhancement of arterial wall was observed on DCE-MRI (Fig. 1). In the active group, DCE-MRI demonstrated the delayed enhancement of arterial wall (Fig. 2) including 3 cases in aorta and its branches, whereas the other 12 cases in both aorta and PA and their branches. Furthermore, no delayed enhancement of arterial wall was observed in the stable TA group (Fig. 3). Delayed enhancement of arterial wall in the active TA group was more than the control group (χ^2^ = 42.00, *P* < .001) and the stable group (χ^2^ = 27.00, *P* < .001).

Arterial stenosis and delayed enhancement are documented in Table 4. On MRA, 32 and 43 stenotic arteries respectively were shown in the active and stable TA patients. The stenotic arteries in the stable TA group was more than those in the active TA group (χ^2^ = 12.373, *P* = .031). DCE-MRI demonstrated 81 arteries with delayed enhancement in the active TA group and none in the stable TA group. In comparison with the stable TA group, delayed enhancement of arterial walls are more in the active TA group (χ^2^ = 27.00, *P* < .001). Moreover, arteries with delayed enhancement are more than stenotic arteries in the active TA group (*Z* = 3.745, *P* = .001).

**Table 4:**
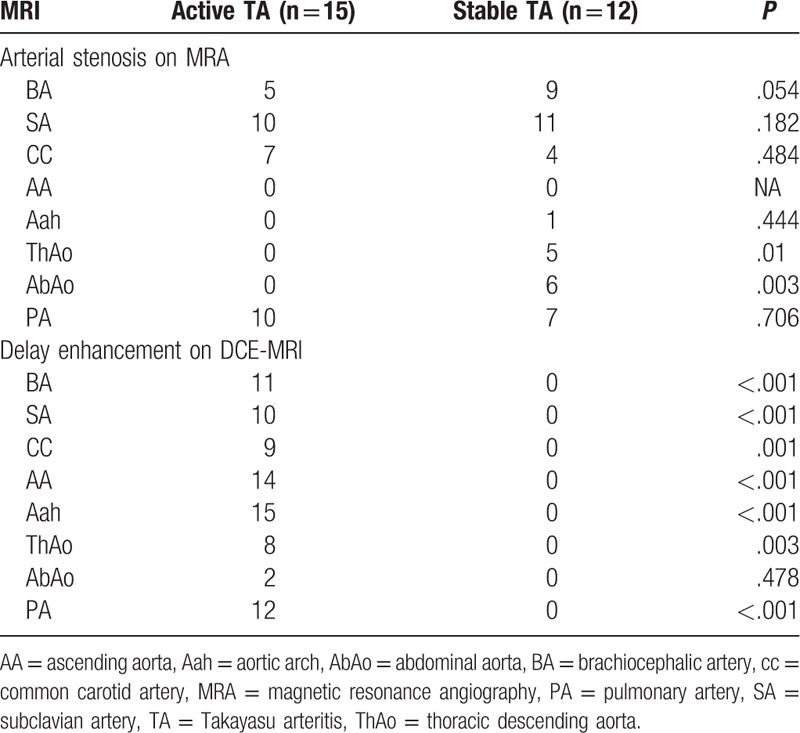
Stenosis and delay enhancement of arteries in patients with Takayasu arteritis.

### 3.3 Pulmonary artery

On MRA, the stenotic PA was observed in 10 active and 7 stable TA patients. Stenotic PA were similar in the active and the stable group (χ^2^ = 0.199, *P* = .656). On DCE-MRI, delayed enhancement of PA was observed in 12 active TA cases. However, no delayed enhancement of PA was observed in the stable group. Compared with the stable TA group, delayed enhancement of PA were more in the active TA group (Fisher exact test, *P* < .001).

On MRA, 11 patients in the active group showed stenosis in right PA (RPA). On DCE-MRI, 2 patients showed the delayed enhancement of RPA, 5 patients showed delayed enhancement of MPA and RPA, and 5 patients showed delayed enhancement of MPA and bilateral PA. PA with delayed enhancement were more than the stenotic PA (χ^2^ = 10.909, *P* = .012).

## 4 Discussion

In this research, we demonstrated that delayed enhancement of arterial wall could be displayed by the free-breathing 3D IR Turbo FLASH sequence in patient with active TA, and delayed enhancement of arterial wall was the main finding of the active TA.

TA is a rare inflammatory disease mostly affecting young women. In our patients, 96.3% (26/27) patients with TA were women and there were no significantly difference of sex and age between the active and stable group. In comparison of the active group, the plasma CRP and ESR in the stable group decreased but NT-proBNP increased. This is related to right ventricular dysfunction and pulmonary hypertension secondary to TA.

TA is characterized by granulomatous inflammation of the arterial wall with marked intimal proliferation and fibrosis of the media and adventitia, which eventually leads to stenosis or occlusion. Wall edema and thickening is prior to the development of luminal stenosis. In principle, MRA acquired with 3D time of flight or 3D spoiled gradient recalled echo T1WI could clearly describe the vessel structure and lumen situation with strongly suppression of the signal of surrounding tissue.^[14–17]^ MRA may be normal in cases of mural thickening without any luminal changes which can be observed in the early stage. In our study, TA was accurately diagnosed by MRA, but the stenotic arteries between the active and stable TA were similar. This means MRA alone is limited to assess the activity of TA. Desai et al^[18]^ showed patient with active TA had evidence of arterial delay enhancement on DCE-MRI and suggest DCE-MRI might be a useful technique to identify inflammation and/or fibrosis in arterial wall. Ginde et al^[19]^ reported in a case, delayed enhancement of the aortic wall correlated with direct histological evidence of inflammation in a girl with the active TA. In our cases, arterial delayed enhancement on 3D IR Turbo FLASH were found in the active TA patients and arteries with the delayed enhancement were more than stenotic arteries. These suggest DCE-MRI with 3D IR Turbo FLASH may detect inflammation before the development of luminal stenosis. The delayed enhancement can be more easily demonstrated at 2D fat-suppressed T1-weighted imaging.^[20]^ Compared with 2D sequence, 3D breath-free IR FLASH sequence can cover the whole aorta in 1 single acquisition with attractive advantages including contiguous sections allowing multiplanar reconstruction in any desirable axis^[8]^ and also can depict of luminal situation and delayed enhancement of the vessel wall. The 3D images can identify the delayed enhancement more conveniently in an arbitrary reformat. This technique could be more suitable for patients with incompliant breath holding and improve patient tolerance. Schneeweis et al^[7]^ report the delayed enhancement of coronary artery wall with 3D IR Turbo FLASH seems to be common in patients with TA, However, they did not observe the delayed enhancement in aorta and its other main branches. In our active TA group, 3D IR Turbo FLASH can depict luminal stenosis and delay enhancement of arterial wall, moreover, arteries with LGE was more than stenotic arteries. These support delayed enhancement of arterial wall with 3D IR Turbo FLASH could a marker of active inflammation of TA.

It was reported^[17]^ that abnormal pulmonary angiography in patients with TA was about 30% to 74%. But Pulmonary artery involvement is often underestimated because pulmonary angiography is not a clinical routine examination. In our case, PA was affected in 52% cases (14/27). PA with delayed enhancement was more than the stenotic PA, suggesting the affected PA depicted with 3D IR Turbo FLASH is more sensitive than MRA. RPA was often involved on MRA; however, delayed enhancement was founded in MPA and bilateral PA on DCE MRI with 3D IR Turbo FLASH. We speculate PA could be one of main targets of TA. This study has some limitations that should be acknowledged. First, this study was a single-center study and included a relatively limited number of TA patients. T2-weighted multiplanar imaging is recommended to evaluate the wall thickening and edema^[21]^; however, we do not assess arterial walls with T2WI because it is time consuming to scan multiple lesions of each patient. Although Desai et al^[18]^ demonstrated the delayed enhancement on DCE-MRI was associated with a raised CRP, in our research, the delayed enhancement on DCE-MRI with IR Turbo FLASH sequence cannot be quantitatively measured, so evaluation of its relation with laboratory findings was impossible. Although renal artery and coronary artery, segmental PA may be involved in TA, we did not include them in this research for its limited spatial resolution.

In conclusion, DCE-MRI with free-breathing three-dimensional inversion recovery Turbo FLASH could monitor the active TA.
